# Activation of the cytosolic calcium-independent phospholipase A_2_ β isoform contributes to TRPC6 externalization *via* release of arachidonic acid

**DOI:** 10.1016/j.jbc.2021.101180

**Published:** 2021-09-10

**Authors:** Priya Putta, Andrew H. Smith, Pinaki Chaudhuri, Rocio Guardia-Wolff, Michael A. Rosenbaum, Linda M. Graham

**Affiliations:** 1 Department of Biomedical Engineering, Cleveland Clinic, Cleveland, Ohio, USA; 2 Department of Vascular Surgery, Cleveland Clinic, Cleveland, Ohio, USA; 3 Surgical Service, Louis Stokes Cleveland Veterans Affairs Medical Center, Cleveland, Ohio, USA

**Keywords:** endothelial cell, canonical transient receptor potential 6 channel (TRPC6 channel), migration, calcium channel, phospholipase A_2_, lysophosphatidylcholine, [Ca^2+^]_i_, intracellular calcium ion concentration, AA, arachidonic acid, BAECs, bovine aortic ECs, cPLA_2_, cytosolic calcium-dependent PLA_2_, EC, endothelial cell, FBS, fetal bovine serum, HPA, heneicosapentaenoic acid, iPLA_2_, cytosolic calcium-independent PLA_2_, lysoPC, lysophosphatidylcholine, NsiRNA, negative control siRNA, oxLDL, oxidized low-density lipoprotein, PLA_2_, phospholipase A_2_, TRPC, canonical transient receptor potential

## Abstract

During vascular interventions, oxidized low-density lipoprotein and lysophosphatidylcholine (lysoPC) accumulate at the site of arterial injury, inhibiting endothelial cell (EC) migration and arterial healing. LysoPC activates canonical transient receptor potential 6 (TRPC6) channels, leading to a prolonged increase in intracellular calcium ion concentration that inhibits EC migration. However, an initial increase in intracellular calcium ion concentration is required to activate TRPC6, and this mechanism remains elusive. We hypothesized that lysoPC activates the lipid-cleaving enzyme phospholipase A_2_ (PLA_2_), which releases arachidonic acid (AA) from the cellular membrane to open arachidonate-regulated calcium channels, allowing calcium influx that promotes externalization and activation of TRPC6 channels. The focus of this study was to identify the roles of calcium-dependent and/or calcium-independent PLA_2_ in lysoPC-induced TRPC6 externalization. We show that lysoPC induced PLA_2_ enzymatic activity and caused AA release in bovine aortic ECs. To identify the specific subgroup and the isoform(s) of PLA_2_ involved in lysoPC-induced TRPC6 activation, transient knockdown studies were performed in the human endothelial cell line EA.hy926 using siRNA to inhibit the expression of genes encoding cPLA_2_α, cPLA_2_γ, iPLA_2_β, or iPLA_2_γ. Downregulation of the β isoform of iPLA_2_ blocked lysoPC-induced release of AA from EC membranes and TRPC6 externalization, as well as preserved EC migration in the presence of lysoPC. We propose that blocking TRPC6 activation and promoting endothelial healing could improve the outcomes for patients undergoing cardiovascular interventions.

Endothelial cell (EC) healing is crucial for successful vascular interventions (1, 2, 3). Oxidized low-density lipoprotein (oxLDL) accumulates at the site of arterial injury caused by vascular interventions and inhibits EC migration. The major component of oxLDL that accounts for its antimigratory property is lysophosphatidylcholine (lysoPC) (4). We have previously shown that lysoPC inhibits EC migration *in vitro* (5), and hypercholesterolemia inhibits EC healing of arterial injuries *in vivo* (6). One of the mechanisms involved in inhibition of EC migration/healing is the activation of canonical transient receptor potential (TRPC) channels, specifically TRPC6, and the subsequent prolonged increase in intracellular calcium ion concentration ([Ca^2+^]_i_) (7, 8). A transient increase in the [Ca^2+^]_i_ is essential to initiate EC migration (9, 10). However, the sustained increase in [Ca^2+^]_i_ specifically due to TRPC6 to TRPC5 channel activation cascade (11) disrupts EC focal adhesions and cytoskeleton that regulate cell movement, thus impeding EC migration essential for injury repair (5). In a mouse arterial injury model, a high-cholesterol diet significantly impairs endothelial healing in WT mice but is not inhibitory in TRPC6 null mice (6). This suggests that blocking lipid oxidation product(s)-induced TRPC6 activation could promote more rapid EC healing leading to improved outcomes after vascular interventions.

TRPC6 channel translocation (*i.e.*, externalization) to the plasma membrane is an essential step that proceeds TRPC6 channel activation. Our previous studies suggest that lysoPC causes an initial local increase in [Ca^2+^]_i_ that is essential to activate TRPC6 channels (8). However, the mechanism by which lysoPC activates TRPC6 and, more specifically, the mechanism of lysoPC-induced TRPC6 externalization to the plasma membrane still remain unclear. LysoPC can activate phospholipase A_2_ (PLA_2_) to release arachidonic acid (AA) from EC membranes (12, 13). This AA can activate arachidonate-regulated calcium channels in the plasma membrane (14), and the subsequent Ca^2+^ entry can provide the local increase in [Ca^2+^]_i_ required to externalize TRPC6 channels. Our working hypothesis is outlined in Figure 1. Currently, no TRPC6 inhibitors are available for clinical use, but inhibiting PLA_2_ activity could potentially block the lysoPC-induced TRPC6 externalization and, therefore, block the activation pathway.

**Figure 1:**
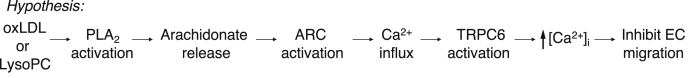
**Schematic of our working hypothesis.** We hypothesize that lysoPC activates phospholipase A_2_ (PLA_2_), which releases arachidonic acid (AA) from the cellular membrane to open arachidonate-regulated calcium (ARC) channels allowing calcium influx that causes TRPC6 channel externalization. The cascade of events after TRPC6 externalization in turn inhibits EC migration. EC, endothelial cell; lysoPC, lysophosphatidylcholine; oxLDL, oxidized low-density lipoprotein; TRPC, canonical transient receptor potential.

PLA_2_ is a superfamily of at least 16 groups of enzymes responsible for the breakdown of glycerophospholipids into lysophospholipids and generation of AA required for eicosanoid and prostaglandin synthesis (15, 16). PLA_2_ enzymes are broadly divided into secretory, cytosolic calcium-dependent (group IV or cPLA_2_), and cytosolic calcium-independent (group VI or iPLA_2_) subgroups (16). cPLA_2_ and iPLA_2_ are the two subgroups most abundantly present in ECs (17). These two subgroups are further divided into various isoforms, including cPLA_2_-α, cPLA_2_-β, cPLA_2_-γ, and cPLA_2_-δ, and iPLA_2_-β, iPLA_2_-γ, -iPLA_2_-ζ, -iPLA_2_-η, and -iPLA_2_-δ (15). The cPLA_2_α-isoform (or group IVA) is the most abundant and well-studied cPLA_2_ isoform, and it contains the characteristic calcium-binding C2 domain required for its activation. However, this calcium-binding C2 domain is lacking in the γ isoform of cPLA_2_ (group IVC), thus making cPLA_2_γ the only known calcium-independent group IV cPLA_2_ isoform (15). cPLA_2_α is involved in EC proliferation and cell cycle progression (18, 19). cPLA_2_γ, although present in the heart, skeletal muscle, and cultured synoviocytes, has unclear biological function (20). Among group VI iPLA_2_, the well-described isoforms are iPLA_2_β and iPLA_2_γ, and both are involved in cell proliferation and membrane remodeling, among other functions (21).

The purpose of this study is to identify PLA_2_ subgroup(s) and the specific isoform(s) that contribute to lysoPC-induced TRPC6 externalization and inhibition of EC migration. We show that iPLA_2_β is the primary isoform involved in lysoPC-induced TRPC6 externalization. Inhibiting iPLA_2_β blocks lysoPC-induced AA release from EC membranes, blocks TRPC6 externalization, and preserves EC migration.

## Results

### LysoPC activated phospholipase enzyme activity and caused AA release in bovine aortic ECs

**Figure 2:**
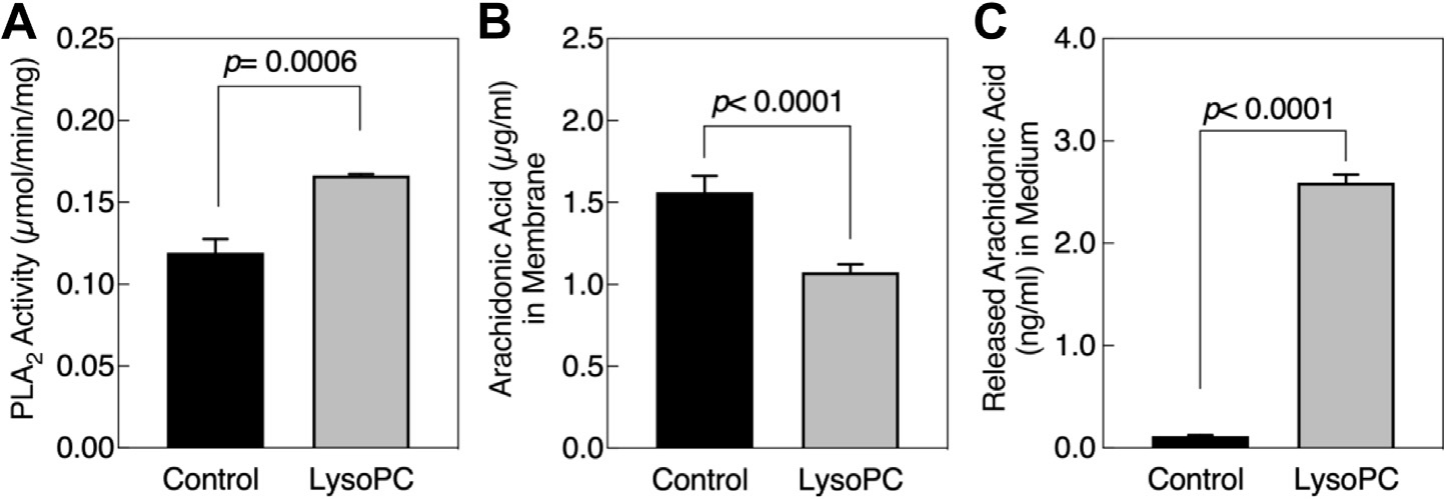
**LysoPC activates phospholipase A**_**2**_**and induces arachidonic acid release from the membrane into the medium.***A*, confluent BAECs were serum-starved for 18 h and then incubated with or without lysoPC (12.5 μM) for 15 min. The cells were then lysed, and the supernatant was assessed for total PLA_2_ enzyme activity. *B* and *C*, confluent BAECs were serum-starved for 18 h and then incubated with or without lysoPC (12.5 μM) for 15 min, and cells were lysed and the membrane fraction isolated. *B*, AA content of the membrane fraction was measured by ELISA. *C*, AA released into the medium was measured by LC/MS/MS. Values shown are the means ± SD (n = 3), analyzed with Student's *t* test and *p* values calculated. BAECs, bovine aortic ECs; lysoPC, lysophosphatidylcholine; PLA_2_, phospholipase A_2_.

To determine if lysoPC activated PLA_2_, PLA_2_ enzyme activity was assessed in bovine aortic ECs (BAECs). ECs were serum-starved for 18 h, and then, 12.5 μM lysoPC was added for 15 min. The cells were lysed, and supernatants were used to determine total PLA_2_ activity with a synthetic substrate, arachidonoyl thio-PC. Under control conditions, PLA_2_ activity was 0.119 ± 0.008 μmol/min/mg (Fig. 2*A*, control). LysoPC increased PLA_2_ activity to 0.166 ± 0.001 μmol/min/mg (Fig. 2*A*, LysoPC), significantly higher than control conditions (n = 3, *p* = 0.0006).

Next, to determine if lysoPC induced release of AA, the AA content of the membrane fraction and the medium was measured. Serum-starved BAECs were incubated with or without 12.5 μM lysoPC for 15 min. The membrane fraction and the medium were isolated to determine the AA content. In the membrane fraction, the AA content was 1.56 ± 0.10 μg/ml in control cells but was reduced to 1.07 ± 0.048 μg/ml in cells incubated with lysoPC (n = 3, *p* < 0.0001, Fig. 2*B*). In contrast, the AA content in the medium was 0.11 ± 0.008 ng/ml in control cells, which increased to 2.59 ± 0.080 ng/ml in cells incubated with lysoPC (n = 3, *p* < 0.0001, Fig. 2*C*). The reduction in the membrane AA content suggested that lysoPC induced the release of AA from the BAEC membranes.

### Downregulation of iPLA_2_*β* but not iPLA_2_γ, cPLA_2_α, or cPLA_2_γ blocked lysoPC-induced TRPC6 externalization

**Figure 3:**
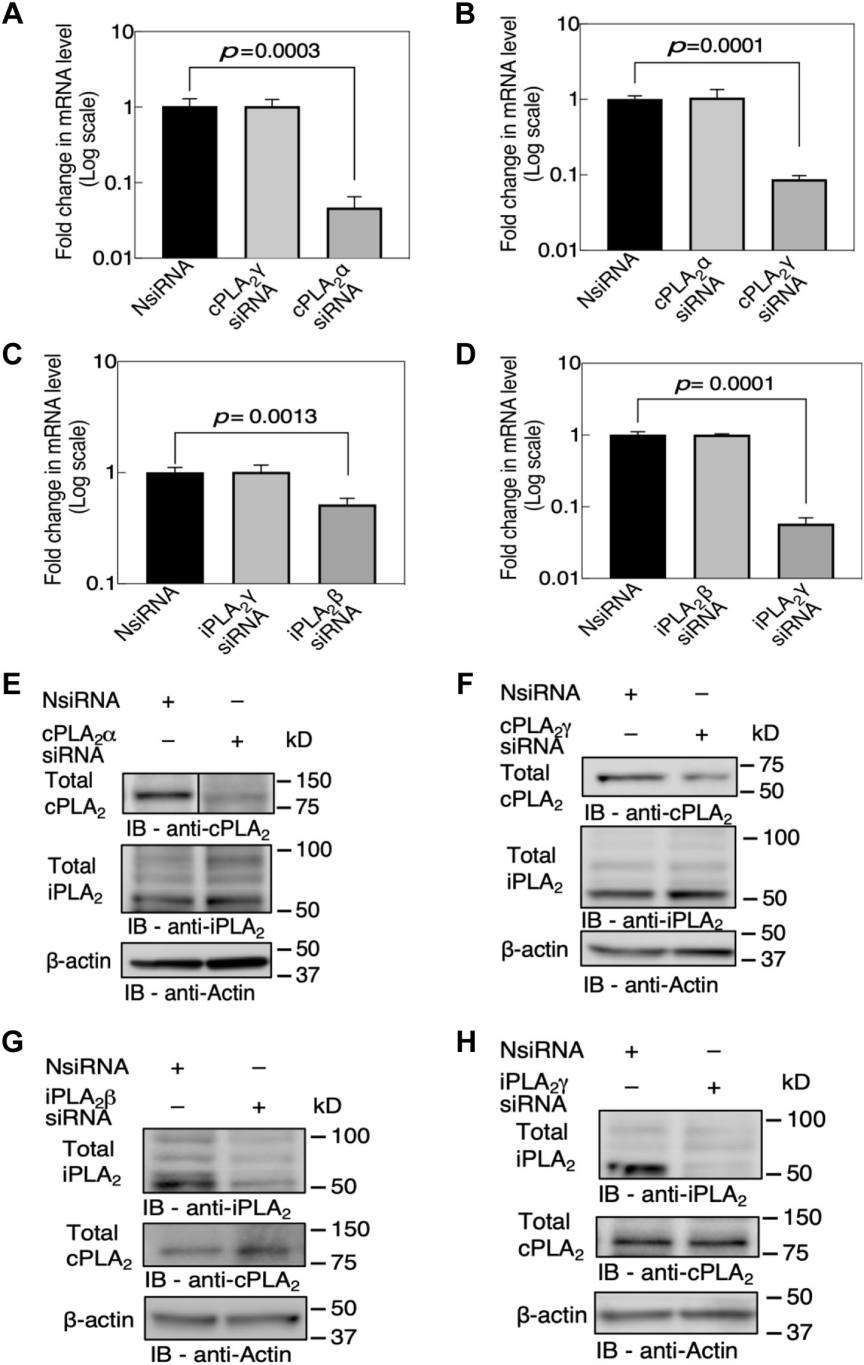
**mRNA and protein expression in siRNA-mediated subgroup-specific PLA**_**2**_**isoform downregulation.** EA.hy926 cells were transiently transfected with 25 nM of control siRNA (NsiRNA) or cPLA_2_α, cPLA_2_γ, iPLA_2_β, or iPLA_2_γ siRNA for 6 h in serum-free medium and then placed in medium with 10% FBS. *A*–*D*, siRNA-mediated downregulation of (*A*) cPLA_2_α, (*B*) cPLA_2_γ (*C*) iPLA_2_β, or (*D*) iPLA_2_γ was quantified using qRT-PCR at 48 h. Values shown are the means ± SD (n = 3), analyzed with one-way ANOVA using Tukey’s multiple comparison test, and *p* values were calculated. *E* and *F*, representative immunoblots depict siRNA-mediated downregulation of PLA_2_ isoforms (*E*) cPLA_2_α, (*F*) cPLA_2_γ (*G*) iPLA_2_β, and (*H*) iPLA_2_γ, detected using subgroup-specific antibody at 48 h after initiation of transfection (n = 3). *Line* in panel *E* indicates lanes rearranged from the same gel. FBS, fetal bovine serum; PLA_2_, phospholipase A_2_.

To identify the role of cPLA_2_ or iPLA_2_ and the specific isoform(s) involved in lysoPC-induced TRPC6 externalization, siRNA-mediated downregulation of PLA_2_ isoforms was undertaken. EA.hy926 cells, a human umbilical vein cell line, were transiently transfected with 25 nmol of cPLA_2_α siRNA, cPLA_2_γ siRNA, iPLA_2_β siRNA, or iPLA_2_γ siRNA. This resulted in a significant decrease in the mRNA levels of the respective isoforms in the siRNA-transfected cells compared with the negative control siRNA (NsiRNA)-transfected cells (n = 3; Fig. 3, *A*–*D*). cPLA_2_α and γ mRNA levels decreased by 96% (n = 3, *p* = 0.0003, Fig. 3*A*) and 91% (n = 3, *p* = 0.0001, Fig. 3*B*), respectively. iPLA_2_β and γ mRNA levels decreased by 78% (n = 3, *p* = 0.0014, Fig. 3*C*) and 96% (n = 3, *p* = 0.0001, Fig. 3*D*), respectively. Downregulation of one isoform did not significantly affect the mRNA expression of the other isoform of the same subgroup (Fig. 3, *A*–*D*).

The decrease in mRNA levels corresponded to a decrease in the protein levels (Fig. 3, *E*–*H*). cPLA_2_ band at ∼90 kD, representing the α-isoform, was present in the NsiRNA-transfected cells and was significantly attenuated in the cPLA_2_α siRNA-transfected cells (Fig. 3*E*). Similarly, cPLA_2_ band at ∼60 kD, representing the cPLA_2_γ isoform, was present in the NsiRNA-transfected cells and was significantly attenuated in the cPLA_2_γ siRNA-transfected cells (Fig. 3*F*). Two distinct bands for iPLA_2_, at ∼90 kDa and ∼63 kDa, were seen in the NsiRNA-transfected cells and were significantly attenuated in both iPLA_2_β and iPLA_2_γ siRNA-transfected cells (Fig. 3, *G* and *H*). Downregulation of one subgroup did not affect the protein expression of the other subgroup (Fig. 3, *E*–*H*).

**Figure 4:**
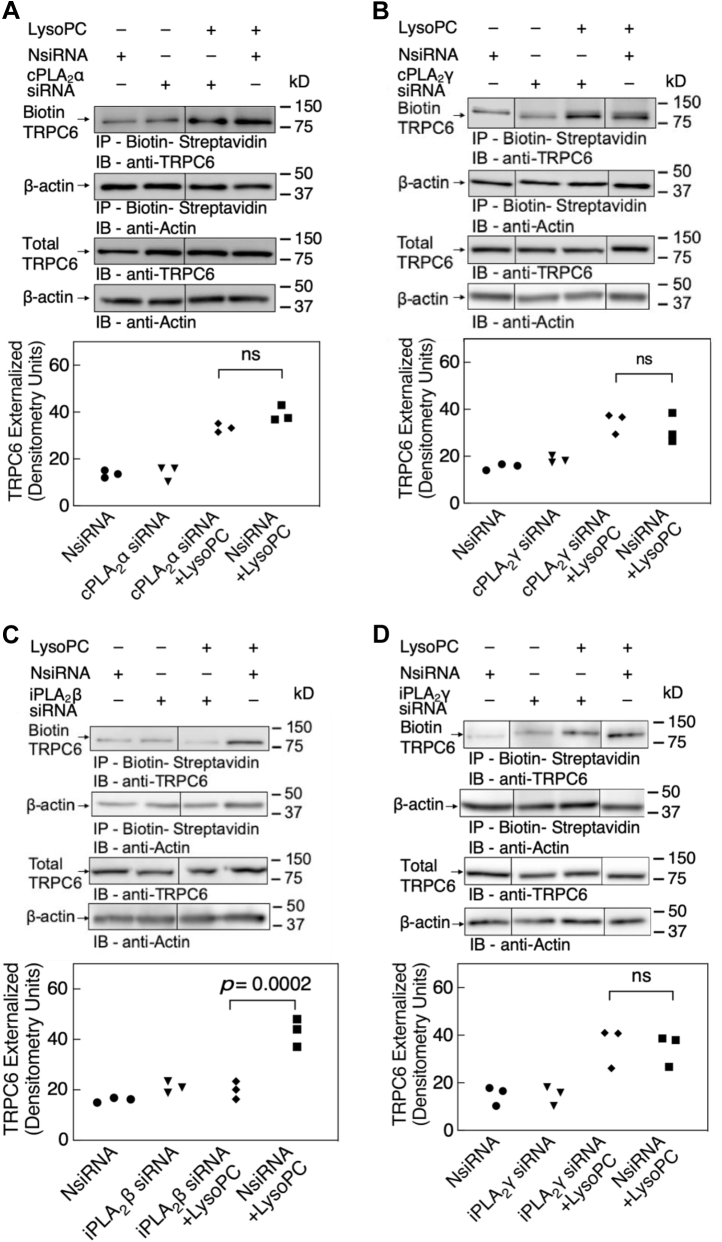
**Downregulation of iPLA**_**2**_**β isoform blocks lysoPC-induced TRPC6 externalization.***A*–*D*, ECs were transiently transfected with NsiRNA or isoform-specific siRNA and serum-starved for 6 h. Then, lysoPC (10 μM) was added for 15 min, and externalized TRPC6 was detected by biotinylation assay. Total TRPC6 was detected in an aliquot of the cell lysate removed before biotinylation, and actin served as a loading control. Representative blots are shown in panel (*A*) cPLA_2_α, (*B*) cPLA_2_γ, (*C*) iPLA_2_β, and (*D*) iPLA_2_γ. *Lines* indicate lanes rearranged from the same gel. Densitometric measurements of externalized TRPC6 are represented in graphic form (n = 3), analyzed with one-way ANOVA using Tukey’s multiple comparison test, and *p* values were calculated. NsiRNA (•); cPLA_2_α, cPLA_2_γ, iPLA_2_β, or iPLA_2_γ siRNA (▾); cPLA_2_α, cPLA_2_γ, iPLA_2_β, or iPLA_2_γ siRNA + lysoPC (♦); NsiRNA + lysoPC (▪). iPLA_2_, cytosolic calcium-independent PLA_2_; lysoPC, lysophosphatidylcholine; ns, not significant; NsiRNA, negative control siRNA; PLA_2_, phospholipase A_2_; TRPC, canonical transient receptor potential.

The effect of cPLA_2_α, cPLA_2_γ, iPLA_2_β, or iPLA_2_γ downregulation on TRPC6 externalization was assessed by biotinylation assay in transfected EA.hy926 cells (Fig. 4, *A*–*D*). At baseline, externalized TRPC6 was comparable for control (NsiRNA), cPLA_2_α, cPLA_2_γ, iPLA_2_β, or iPLA_2_γ siRNA-transfected cells. Incubation with 10 μM lysoPC for 15 min increased TRPC6 externalization in ECs transfected with NsiRNA. Downregulation of cPLA_2_α did not result in a significant decrease in lysoPC-induced TRPC6 externalization (n = 3; *p* = 0.057 compared with NsiRNA with lysoPC, Fig. 4*A*). Similarly, downregulation of cPLA_2_γ isoform did not block lysoPC-induced TRPC6 externalization (n = 3; *p* = 0.53 compared with NsiRNA with lysoPC, Fig. 4*B*). Interestingly, the lysoPC-induced TRPC6 externalization was significantly attenuated in iPLA_2_β downregulated cells compared with NsiRNA with lysoPC (n = 3, *p* < 0.003, Fig. 4*C*). Transient knockdown of iPLA_2_γ, however, did not alter the lysoPC-induced TRPC6 externalization (n = 3; *p* > 0.8 compared with NsiRNA with lysoPC, Fig. 4*D*). These results suggested that lysoPC primarily activated iPLA_2_β to promote externalization of TRPC6.

### Downregulation of iPLA_2_*β* and not iPLA_2_γ, cPLA_2_α, or cPLA_2_γ blocked lysoPC-induced inhibition of EC migration

**Figure 5:**
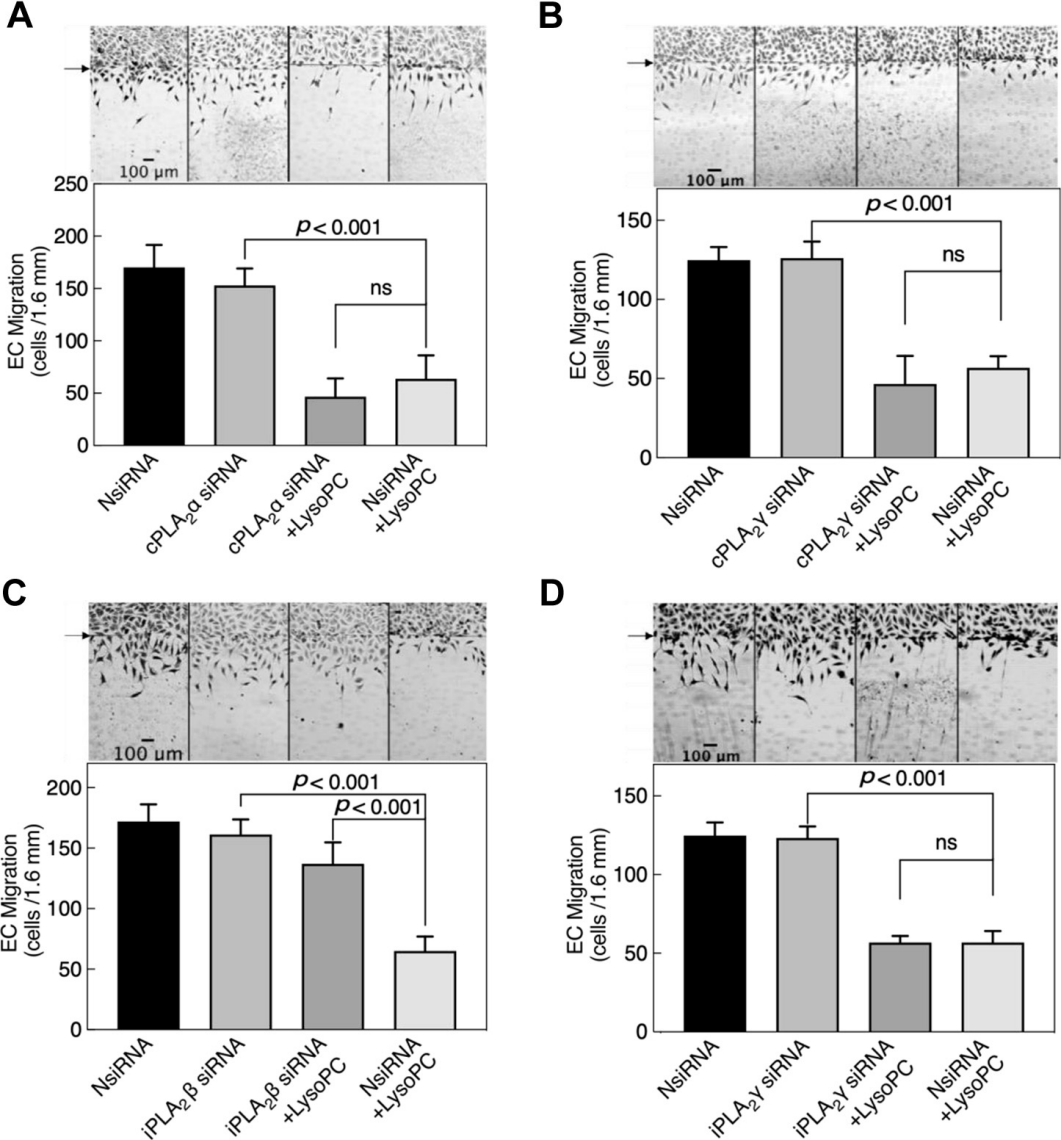
**Downregulation of iPLA**_**2**_**β isoform preserves EC migration in the presence of lysoPC.***A*–*D*, ECs were transiently transfected with NsiRNA or isoform-specific siRNA and then serum-starved for 6 h. The migration assay was initiated ± lysoPC (10 μM). Migration was quantified at 24 h. The *arrow* indicates the starting line of cell migration for assessment of the effect of (*A*) cPLA_2_α, and (*B*) cPLA_2_γ, (*C*) iPLA_2_β, or (*D*) iPLA_2_γ downregulation. The graphs represent mean ± SD (n = 4), analyzed with one-way ANOVA using Tukey’s multiple comparison test, and *p* values were calculated. Representative images of four experiments are shown, 40× magnification; the scale bar represents 100 μm. cPLA_2_, cytosolic calcium-dependent PLA_2_; EC, endothelial cell; iPLA_2_, cytosolic calcium-independent PLA_2_; lysoPC, lysophosphatidylcholine; ns, not significant; NsiRNA, negative control siRNA.

The effect of cPLA_2_α, cPLA_2_γ, iPLA_2_β, or iPLA_2_γ downregulation on lysoPC-induced inhibition of EC migration was assessed by razor scrape assay in EA.hy926 transfected cells (Fig. 5). Basal EC migration for NsiRNA, cPLA_2_α siRNA, cPLA_2_γ siRNA, iPLA_2_β siRNA, or iPLA_2_γ siRNA-transfected cells was similar (Fig. 5, *A*–*D*). In ECs transfected with NsiRNA, lysoPC reduced migration by ∼55 to 60% (n = 3; *p* < 0.001, Fig. 5, *A*–*D*). In cPLA_2_α siRNA-transfected ECs, lysoPC inhibited migration by ∼70% (n = 3; *p* = 0.44, comparable with NsiRNA with lysoPC, Fig. 5*A*). Similarly, in cPLA_2_γ siRNA-transfected ECs, lysoPC inhibited migration by ∼63% (n = 3; *p* = 0.47 comparable with NsiRNA with lysoPC, Fig. 5*B*). However, lysoPC inhibited migration by only 15% in iPLA_2_β downregulated cells (n = 3; *p* < 0.001 compared with NsiRNA with lysoPC, Fig. 5*C*). LysoPC continued to inhibit migration in iPLA_2_γ downregulated ECs, as it did in NsiRNA-transfected EC with lysoPC (n = 3; *p* > 0.9, Fig. 5*D*). Individual siRNAs were studied to determine if the effect on migration was due to off-target effect of pooled iPLA_2_β siRNA. The individual iPLA_2_β siRNAs (35 nM) showed similar effects compared with the pooled iPLA_2_β siRNA in preserving EC migration in lysoPC (Fig. S1, *A* and *B*). These results suggested that lysoPC activated iPLA_2_β to inhibit EC migration in an *in vitro* migration assay.

### Downregulation of iPLA_2_β inhibited lysoPC-induced release of AA

**Figure 6:**
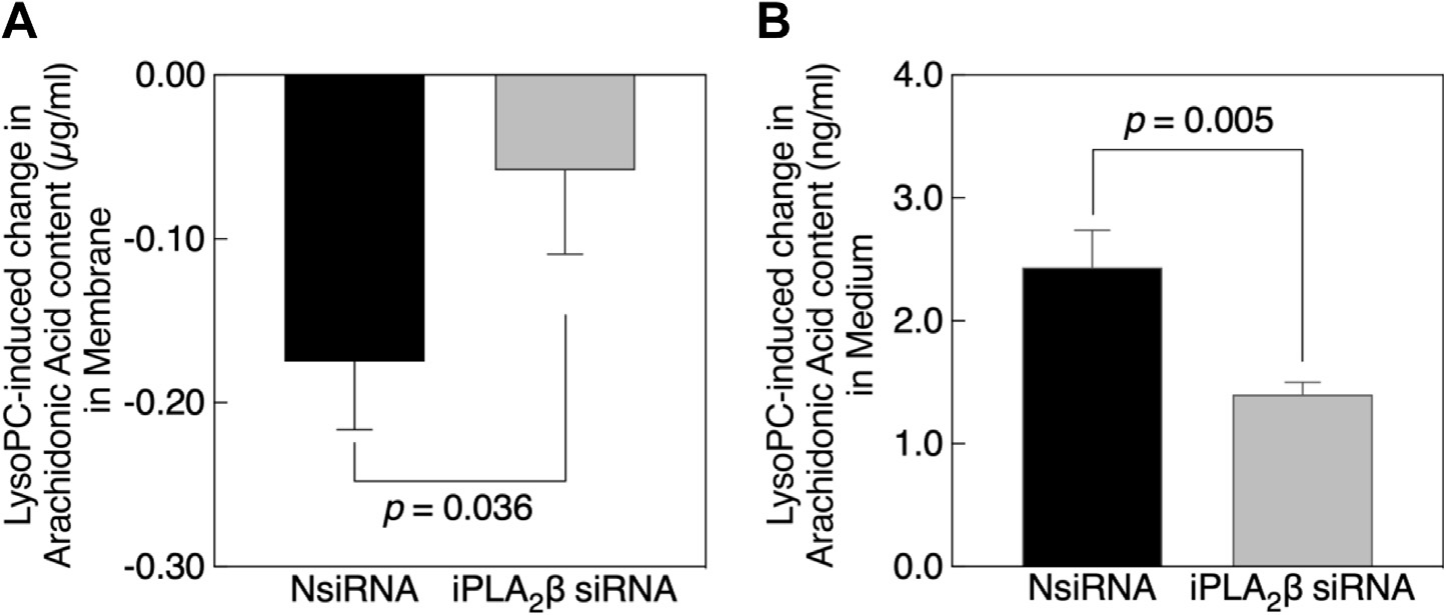
**Downregulation of iPLA**_**2**_**β isoform blocks lysoPC-induced arachidonic acid release from the membrane into medium.***A* and *B*, ECs transfected with NsiRNA or iPLA_2_β siRNA were serum-starved for 6 h, then lysoPC (10 μM) added for 15 min. Cells were lysed, and the AA content of the membrane fraction was measured by ELISA, and the AA content in the medium was measured by LC/MS/MS. *A*, lysoPC-induced change in AA content in membrane fraction. *B*, lysoPC-induced change in AA content in the medium. Values shown are the means ± SD (n = 3), analyzed with Student's *t* test and *p* values calculated. EC, endothelial cell; iPLA_2_, cytosolic calcium-independent PLA_2_; lysoPC, lysophosphatidylcholine; NsiRNA, negative control siRNA.

To ascertain if downregulation of iPLA_2_β prevented the lysoPC-induced AA release from the membrane, AA ELISA assay was performed in transfected EA.hy926 cells. Cells were incubated with lysoPC for 15 min, then lysed, the membrane and medium fractions were isolated to assess the AA content (Fig. 6). AA content for NsiRNA and iPLA_2_β siRNA transfected cell membranes was similar. LysoPC decreased the AA in the membrane in NsiRNA-transfected cells by 0.175 ± 0.041 μg/ml, but by only 0.0583 ± 0.050 μg/ml in iPLA_2_β siRNA-transfected cells (n = 3, *p* < 0.036, Fig. 6*A*). This confirmed that iPLA_2_β downregulation blocked lysoPC-induced release of AA from EC membranes. Similarly, lysoPC increased the AA content in the medium fraction of NsiRNA-transfected cells by 2.433 ± 0.305 ng/ml, but by only 1.4 ± 0.1 ng/ml in iPLA_2_β siRNA-transfected cells (n = 3, *p* < 0.005, Fig. 6*B*). The prevention of lysoPC-induced AA release from EC membranes in iPLA_2_β downregulated cells could contribute to the preservation of EC migration in the presence of lysoPC.

### Downregulation of iPLA_2_β inhibited lysoPC-induced increase in [Ca^2+^]_i_

**Figure 7:**
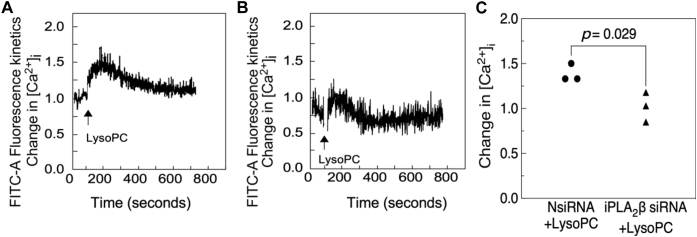
**Downregulation of iPLA**_**2**_**β isoform inhibits lysoPC-induced increase in [Ca**^**2+**^**]**_**i**_**.** ECs transfected with NsiRNA and iPLA_2_β siRNA were serum-starved for 6 h. ECs were loaded with the FITC-conjugated fluorophore Calbryte 520 AM dye. The ECs were suspended and loaded into the sort chamber of a BD FACSMelody Cell Sorter maintained at 37 °C. After adjusting the baseline, lysoPC (10 μM) was added. *A*–*C*, using the kinetic reading mode at Ex/Em 490/525 nm, relative changes in [Ca^2+^]_i_ after transfection with (*A*) NsiRNA or (*B*) iPLA_2_β siRNA were determined. Representative graphs of three experiments are shown here. *C*, change in [Ca^2+^]_i_ measured by difference in mean [Ca^2+^]_i_ at baseline and after addition of lysoPC is presented in the graph. Values shown are the means ± SD (n = 3), analyzed with Student's *t* test and *p* values calculated. iPLA_2_, cytosolic calcium-independent PLA_2_; lysoPC, lysophosphatidylcholine; EC, endothelial cell; NsiRNA, negative control siRNA.

We evaluated if downregulation of iPLA_2_β prevented the lysoPC-induced increase in [Ca^2+^]_i_ using fluorometric assay. EA.hy926 cells transfected with NsiRNA or iPLA_2_β siRNA were loaded with the FITC-conjugated fluorophore Calbryte 520 AM dye. The ECs were suspended and loaded into the sort chamber of a BD FACSMelody Cell Sorter maintained at 37 °C. After adjusting the baseline, lysoPC (10 μM) was added and the change in [Ca^2+^]_i_ was recorded. LysoPC increased [Ca^2+^]_i_ in NsiRNA-transfected cells to 1.38 times the baseline (representative graph, Fig. 7*A*), but only to 1.02 times in iPLA_2_β siRNA-transfected cells (representative graph, Fig. 7*B*). iPLA_2_β siRNA-transfected cells significantly attenuated lysoPC-induced increase in [Ca^2+^]_i_ (n = 3, *p* < 0.029, Fig. 7*C*). These results supported the role of iPLA_2_β in lysoPC-induced increase in [Ca^2+^]_i_ required for TRPC6 externalization and inhibition of EC migration.

## Discussion

OxLDL and lysoPC inhibit EC migration, and there is sufficient lysoPC in oxLDL to account for its antimigratory activity (4). LysoPC is one of the most potent antimigratory lysophospholipids, and our previous studies have shown that it inhibits EC migration at least in part by activating TRPC6, which leads to a cascade of events resulting in a prolonged increase in [Ca^2+^]_i_ that activates calpains and inhibits cytoskeletal changes required for migration (5, 11). LysoPC-induced TRPC6 externalization requires a small, perhaps localized, increase in [Ca^2+^]_i_ (8); however, the source of the initial lysoPC-induced calcium flux in ECs is unclear. We postulate that lysoPC activates PLA_2_ causing release of AA, which in turn opens arachidonate-regulated calcium channels leading to the localized increase in calcium. The goal of this study is to identify the PLA_2_ involved in lysoPC-induced TRPC6 externalization and activation leading to inhibition of EC migration. The results presented here demonstrate that lysoPC activates PLA_2_, in keeping with the findings of Lupo *et al.* (22) using rat brain ECs and oxLDLs. We also show that lysoPC releases AA from the cell membrane, which is in agreement with Wong *et al.* (12) who showed lysoPC induces AA release in human umbilical vein ECs. Interestingly, we show that iPLA_2_, but not cPLA_2_, mediates lysoPC-induced TRPC6 externalization. Using siRNA-mediated downregulation of specific isoforms, the β-isoform, but not the γ-isoform, of iPLA_2_ appears to be responsible for lysoPC-induced TRPC6 externalization. Downregulating iPLA_2_β inhibits lysoPC-induced release of AA from the EC membrane, blocks the increase in [Ca^2+^]_i_, and preserves EC migration in the presence of lysoPC.

Earlier studies suggest that cPLA_2_ is the major isoform involved in the AA release from membranes, whereas iPLA_2_ is a housekeeping protein only involved in the incorporation of free AA into membranes (23, 24, 25). However, later studies show that in addition to its housekeeping function, iPLA_2_ is involved in signal transduction pathways, as well as generation of AA and other lipid metabolites (26, 27). In fact, a role for iPLA_2_ is suggested in agonist-induced AA release in aortic smooth muscle cells and RAW 264.7 macrophage cell line (28, 29). Furthermore, Balboa and Balsinde demonstrate the key role of iPLA_2_ in the release of AA in human U937 cells during oxidative stress (30, 31). In addition, the role of iPLA_2_β is associated with thrombin-induced AA release in human coronary artery ECs (32). Our data are consistent with these studies and suggest a role for iPLA_2_, specifically the β isoform of iPLA_2,_ in mediating lysoPC-induced TRPC6 externalization and activation in ECs.

Downregulating iPLA_2_β modestly inhibits lysoPC-induced release of AA from the EC membrane (Fig. 6) but significantly preserves EC migration in the presence of lysoPC (Fig. 5*C*). LysoPC-induced AA release from the membrane is a localized event. Upon release, the free AA is either rapidly metabolized or incorporated back into phospholipid pool, or diffused into other cells (33). Wong *et al.* (12) show that AA release in ECs is both time and concentration dependent, maximal AA release being observed at 10 min with 50 μM lysoPC. In our AA release assay, 10 μM lysoPC is used to align with our functional assays and our previous studies. Hence, we observe the modest difference between lysoPC-induced AA release in cells transfected with NsiRNA compared with iPLA_2_β siRNA. Furthermore, using the razor scrape assay, we demonstrate that this modest difference in AA release translates into prevention of lysoPC-induced inhibition of EC migration in iPLA_2_β-downregulated cells (Fig. 5*C*). AA release is measured minutes after incubation with lysoPC, while migration is measured at 24 h, allowing for a series of events to occur. The robust effect on migration in iPLA_2_β downregulated cells may reflect the efficacy of AA release inhibition which is upstream in a cascade of events that eventually result in cytoskeletal changes that block migration.

iPLA_2_β has been shown to be involved in the activation of other TRP channels such as TRPC5 (34) and TRPM8 (35). AL-Shawaf *et al.* (34) show that downregulation of iPLA_2_β suppresses sphingosine 1-phosphate–induced, but not lysoPC-induced, TRPC5 channel activation in HEK cells containing conditional expression of TRPC5 (34). Our previous studies in ECs expressing both TRPC6 and TRPC5 show that lysoPC-induced TRPC6 activation precedes TRPC5 activation and that downregulation of TRPC6 suppresses TRPC5 externalization in ECs incubated with lysoPC (11). In our present study, blocking iPLA_2_β inhibits lysoPC-induced TRPC6 externalization, which should result in decreased TRPC5 activation in cells expressing both channels. The difference in the role of iPLA_2_β in the TRPC activation in these studies may reflect the variety of mechanisms for TRPC5 activation, including activation by reactive oxygen species, changes in [Ca^2+^]_i_, or directly by lysoPC (36, 37).

Oxidized lipid products impede endothelial healing during vascular interventions. We have shown previously that lysoPC disrupts the delicate balance of [Ca^2+^]_i_ in ECs by activation of TRPC6 and *via* signal transduction pathways leading to TRPC5 activation and inhibition of EC migration (5, 7, 8, 11, 38). Our present study shows for the first time the role of iPLA_2_β in the externalization of TRPC6 and subsequent inhibition of EC migration by lysoPC. These results allow for selection of an isoform-specific pharmacological inhibitor, several of which are currently being used in clinical cancer therapy trials, and to test its efficacy to promote endothelial healing in an arterial injury model. Specifically blocking lysoPC-induced iPLA_2_β activation in ECs should prevent TRPC6 activation and preserve EC migration, thereby improving endothelial healing after interventions for cardiovascular diseases.

## Experimental procedures

### Cells and reagents

BAECs were isolated from adult bovine aortas by scraping after collagenase treatment (11). Assays involving BAECs were performed in replicates using cells from at least three different bovine aortas. BAECs between passages 4 and 9 were used for the assays. EAhy.926 cells, a primary human umbilical vein cell line, were purchased from the ATCC.

1-Palmitol-2-hydroxy-sn-glycero-3-phosphocholine (16:0 LysoPC) (catalog number (#): 855675p) was obtained from Avanti Polar Lipids, Inc AA (#90010) and heneicosapentaenoic acid (HPA) (#10670) were purchased from Cayman Chemical. The PLA_2_ assay kits (#765021) were purchased from Cayman Chemical, and the AA ELISA kits (#MBS2608709 and #MBS267742) were purchased from MyBioSource. Antibodies for immunoblot analysis were purchased from Cell Signaling Technology and Santa Cruz Biotechnology as indicated below. siRNA for transfection studies were purchased from Dharmacon, Inc. ON-TARGETPlus siRNA SMARTpool [PLA2G4A: L-009886-00-0005, PLA2G4C: L-009663-00-0005, PLA2G6: L-009085-00-0005 (J-009085-12-0002 and J-009085-13-0002 individual siRNAs), PLA2G6B: L-010284-00-0005], and the NsiRNA were purchased from Santa Cruz Biotechnology (#sc-37007). RNA isolation kits were purchased from Qiagen (miRNeasy mini kit # 217004) and TaqMan assay kits for qRT-PCR were purchased from Thermo Fisher [PLA2G4A (Hs00233352_m1), PLA2G4C (Hs00234345_m1), PLA2G6A (Hs00899715_m1), PLA2G6B (HS00382272-m1), or GAPDH (Hs99999905_m1)].

### EC culture

BAECs were cultured in Dulbecco’s modified Eagle’s medium containing 10% (vol/vol) fetal bovine serum (FBS, HyClone Laboratories #SH30541.03) and 1% antibiotic (penicillin/streptomycin). EA.hy926 were cultured in Eagle's Modified Essential Medium containing 10% (vol/vol) FBS.

### Measurement of PLA_2_ activity

Total PLA_2_ activity in BAECs was measured using the cPLA_2_ assay kit. The use of this assay kit without the specific purification procedure allowed measurement of total PLA_2_ enzyme activity (39, 40). BAECs were grown in 60-mm dishes and serum-starved for 18 h. LysoPC (12.5 μM) was added for 15 min in appropriate dishes. Cells were then lysed in the lysis buffer (50 mM Hepes, 150 mM NaCl, 200 μM Na_3_VO_4_, 100 mM NaF, 1% Triton X-100, pH 7.4) containing protease inhibitors (cOmplete, Roche) for 30 min at 4 °C. Lysates were passed through needles, 20-gauge (20×) and 25-gauge (15×), and cleared by centrifugation at 12,000*g* for 15 min. PLA_2_ assay was performed as per the manufacturer’s protocol. Briefly, sample, blank, and positive control (bee venom) (10 μl) were added to a 96-well plate in triplicates. To initiate the reaction, arachidonoyl Thio-PC (200 μl) substrate was added to each well and mixed and incubated for 60 min at room temperature. DNTB/EGTA was then added to stop the enzymatic reaction and the absorbance read at 405 nm using a plate reader (SpectraMAX 190).

### Downregulation of PLA_2_

EA.hy926 cells at 70 to 80% confluency were incubated with 25 nM siRNA for 6 h using DharmaFECT reagent in serum-free medium according to the manufacturer’s protocol, followed by full replacement of the medium supplemented with 10% FBS for the remainder of the 48 h. siRNA for PLA2G4A, PLA2G4C, PLA2G6A, and PLA2G6B (Dharmacon, Inc) and NsiRNA (Santa Cruz Biotechnology) were used. mRNA was isolated at 48 h with Qiagen miRNeasy mini kit and knockdown efficiency quantified with RT-qPCR using TaqMan assay kits. Samples were analyzed in triplicate, and target gene expression was normalized to GAPDH. Protein level knockdown was assessed at 48 h with immunoblot analysis.

### Measurement of the AA content in the membrane and medium

BAECs or transfected EA.hy926 cells were serum-starved for 18 h or 6 h, respectively. LysoPC (12.5 μM or 10 μM) was then added for 15 min. Cells were processed as per the manufacturer’s protocol using the Mem-PER Plus membrane extraction kit (Thermo Fisher) to obtain the membrane fraction. Briefly, cells were washed and centrifuged, the pellet was resuspended in the permeabilization buffer (350 μl), incubated for 10 min at 4 °C, and centrifuged at 16,000*g* for 15 min at 4 °C. The cytosolic fraction was carefully separated, the pellet was further resuspended in the solubilization buffer (250 μl) for 30 min at 4 °C, and centrifuged at 16,000*g* for 15 min to collect the membrane fraction. The AA content of the membrane fraction was measured using an AA ELISA kit as per the manufacturer’s protocol and the absorbance read at 450 nm using a plate reader (SpectraMAX 190).

For AA measurement, the medium was collected and processed using HPLC On-line LC/MS/MS. Briefly, the cell medium (500 μl) was mixed with methanol (50 μl) containing 2 μg/ml HPA as the internal standard and dried under N_2_ flow. Methanol 75% (50 μl) was added to the dried sample, vortexed, and then filtered through a 0.22-μm membrane. A 5-μl aliquot was injected to the Vanquish HPLC and Quantiva triple quadrupole mass spectrometer (Thermo Fisher) (41). XCalibur software was used to process the data and obtain the peak areas of AA and HPA. The internal standard calibration curve was used to calculate the concentration of AA in the samples.

### Immunoblot analysis

Immunoblot analysis was performed as previously described (11). Proteins of interest were detected using antibodies specific for rabbit TRPC6 (1:1000, Cell Signaling #16716S), rabbit cPLA_2_ (1:1000, Cell Signaling #2832), mouse iPLA_2_ (1:1000, Santa Cruz Biotechnology #sc-376563), and β-actin (1:2000, Santa Cruz Biotechnology #sc47778 HRP). Anti-rabbit (1:1000, antibodies-online #ABIN102010) or anti-mouse (1:1000, Santa Cruz Biotechnology #SC516102) antibodies were used for secondary antibodies.

### TRPC6 externalization by biotinylation assay

Biotinylation of EC membrane surface proteins was performed as previously described (42). Briefly, transfected EA.hy926 cells were cultured in 60-mm dishes to 80% confluency and serum-starved for 6 h, and then lysoPC (10 μM) was added for 15 min. Externalized TRPC6 was detected by the biotinylation assay (42) and immunoblot analysis was performed.

### EC migration

EC migration was assessed in a razor scrape assay in 12-well tissue culture plates as previously described (43). Briefly, transfected EA.hy926 cells were serum-starved for 6 h. The razor scrape was performed and cells allowed to migrate ± lysoPC (10 μM) for 24 h. Using a digital CCD camera mounted on a phase-contrast microscope, images were taken of three random fields, each corresponding to a starting line length of 1.6 mm, from three wells. Images were processed using NIH ImageJ analysis software (NIH, Bethesda, MD), and an observer blinded to the experimental conditions quantitated the migration.

### Measurement of [Ca^2+^]_i_

ECs at 80 to 90% confluence were loaded with the FITC132 conjugated fluorophore Calbryte 520 AM dye (AAT Bioquest; Catalog No. 36310) following the manufacturer’s protocol. After 35 min, the EC were suspended and loaded into the sort chamber of a BD FACSMelody Cell Sorter (BD Biosciences) maintained at 37 °C. After adjusting the baseline, 10 μM lysoPC was added and relative change in [Ca^2+^]_i_ was read using the kinetic reading mode at Ex/Em 490/525 nm. Kinetics data were analyzed using the FlowJo v10 software (BD Biosciences).

### Statistics analysis

All experiments were performed at least in triplicate. Studies with BAECs used ECs isolated from at least three different animals. Values are presented as the mean ± SD. Data were analyzed by Student's *t* test or one-way ANOVA with appropriate post hoc analysis, and *p* < 0.05 was considered statistically significant.

## Data availability

All the data described in the article are contained within the article.

## Supporting information

This article contains supporting information.

## Conflict of interest

The authors declare that they have no conflicts of interest with the contents of this article.
