# Supplementary material for: Activation of the cytosolic calcium-independent phospholipase A2 β isoform contributes to TRPC6 externalization via release of arachidonic acid
Source: J Biol Chem. 2021 Sep 10;297(4):101180. doi: 10.1016/j.jbc.2021.101180 (PMC8498464; doi:10.1016/j.jbc.2021.101180)
Supplement: Supplemental Figure S1 [file mmc1.pdf]

Activation of the cytosolic calcium-independent phospholipase A_2 β isoform contributes to TRPC6 externalization via release of arachidonic acid

Priya Putta[1*], Andrew H. Smith[2], Pinaki Chaudhuri[1], Rocio Guardia-Wolff[1], Michael A. Rosenbaum[3], Linda M. Graham[1,2]

[1]Department of Biomedical Engineering, Cleveland Clinic, Cleveland, Ohio 44195.
[2]Department of Vascular Surgery, Cleveland Clinic, Cleveland, Ohio 44195.
[3]Surgical Service, Louis Stokes Cleveland Veterans Affairs Medical Center, Cleveland, OH 44106.

*Corresponding author:
Priya Putta
Email: puttaP@ccf.org

Running title: Role of $iPLA_2$ in TRPC6 externalization

Key words: endothelial cell, canonical transient receptor potential 6 channel (TRPC6 channel), migration, calcium channel, phospholipase A_2, lysophosphatidylcholine

Figure S1.

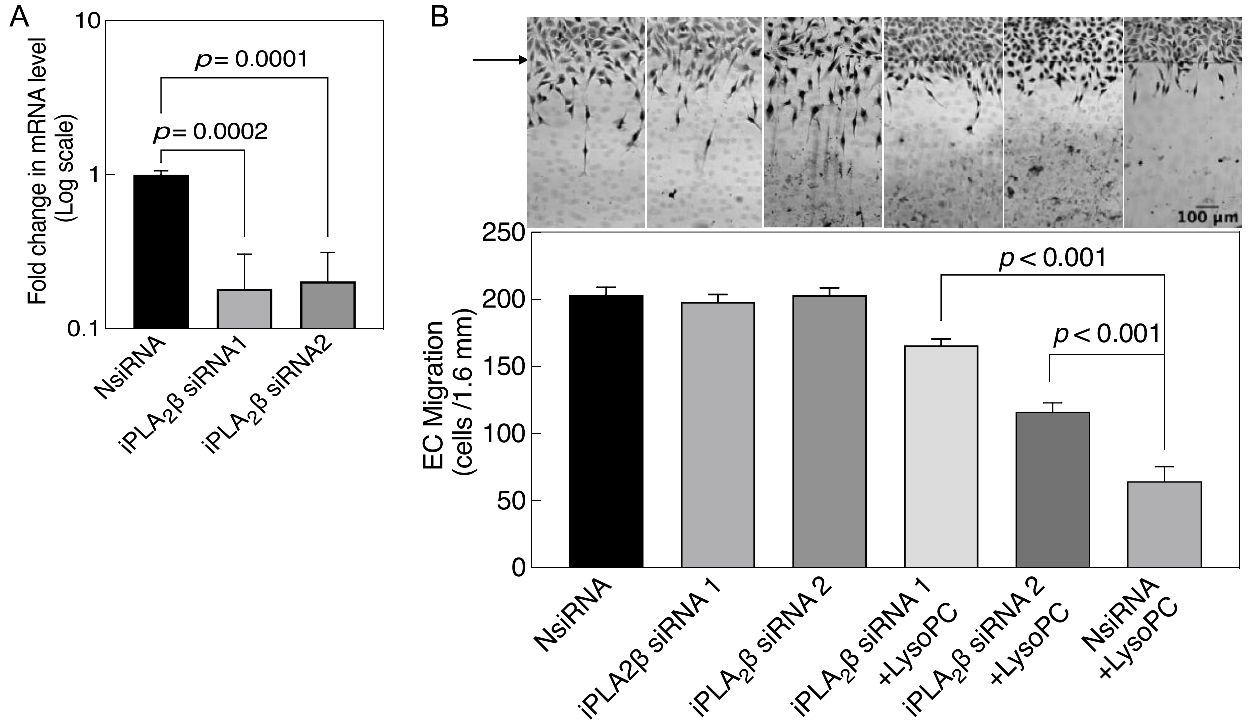

Figure S1. Downregulation of iPLA2β isoform using individual siRNAs preserves EC migration in the presence of lysoPC. ECs were transiently transfected with 35 nM of control NsiRNA, iPLA2β siRNA 1, or iPLA2β siRNA 2, then serum-starved for 6 h. **(A)** siRNA mediated downregulation was confirmed with RT-PCR. **(B)** The migration assay was initiated ± lysoPC (10 µM). Migration was quantified at 24 h. The arrow indicates the starting line of cell migration. The graphs represent mean ± SD of n=3; analyzed with one-way ANOVA using Tukey's multiple comparison test, and *p* values were calculated. Representative images are shown, 40x magnification, scale bar 100 µm.
